# A systematic analysis of a broadly neutralizing antibody AR3C epitopes on Hepatitis C virus E2 envelope glycoprotein and their cross-reactivity

**DOI:** 10.1186/1755-8794-8-S4-S6

**Published:** 2015-12-09

**Authors:** Jing Sun, Vladimir Brusic

**Affiliations:** 1 Dana-Farber Cancer Institute, Harvard Medical School, Boston, MA, USA; 2 School of Medicine and Bioinformatics Center, Nazarbayev University, Astana, Kazakhstan

## Abstract

**Background:**

Hepatitis C virus (HCV) belongs to *Flaviviridae *family of viruses. HCV represents a major challenge to public health since its estimated global prevalence is 2.8% of the world's human population. The design and development of HCV vaccine has been hampered by rapid evolution of viral quasispecies resulting in antibody escape variants. HCV envelope glycoprotein E1 and E2 that mediate fusion and entry of the virus into host cells are primary targets of the host immune responses.

**Results:**

Structural characterization of E2 core protein and a broadly neutralizing antibody AR3C together with E1E2 sequence information enabled the analysis of B-cell epitope variability. The E2 binding site by AR3C and its surrounding area were identified from the crystal structure of E2c-AR3C complex. We clustered HCV strains using the concept of "discontinuous motif/peptide" and classified B-cell epitopes based on their similarity.

**Conclusions:**

The assessment of antibody neutralizing coverage provides insights into potential cross-reactivity of the AR3C neutralizing antibody across a large number of HCV variants.

## Background

Hepatitis C virus (HCV) is a major cause of viral hepatitis, liver cirrhosis, and liver cancer. It was discovered in 1989 as a novel causative agent of hepatitis [1]. HCV is a growing health concern since it affects about 2.8% of the world population and its prevalence is rising [2,3]. Each year, there are more than 500,000 new HCV infections in Egypt, the country with the highest HCV prevalence [4]. In the United States, more people die from HCV than from human immunodeficiency virus 1 (HIV-1) related disease [5]. Six genotypes and multiple subtypes of HCV have been identified to date. Approximately 75% of Americans with HCV have genotype 1 of the virus (subtypes 1a or 1b), and 20-25% have genotypes 2 or 3, with small numbers of patients being infected with genotypes 4, 5, or 6 [6]. Effective vaccination would provide protection against this global disease. However, the development of HCV vaccine and identification of broadly neutralizing antibodies has been hampered because HCV sequences mutate rapidly generating escape variants [7], the non-neutralizing antibodies to HCV envelope proteins interfere with neutralizing antibodies [8], and there is lack of 3D structural information needed for vaccine development [9]. The first crystal structure of broadly neutralizing antibody against HCV has been published in 2013 [10].

The HCV envelope glycoproteins E1 and E2 form a heterodimer E1E2 that facilitates virus attachment and entry into host cells and are targets for neutralizing antibodies [11]. Recent progress in isolating and characterizing HCV-neutralizing antibodies are instrumental for vaccine discovery and design [12]. These HCV-neutralizing antibodies were isolated from immunized mice [13-15], or from patients chronically infected with HCV [16-20]. Giang *et al*. [4], using an exhaustive panning strategy, identified five distinct antigenic regions on the HCV E1E2, that were recognized by 73 human monoclonal antibodies (mAbs) from an HCV immune phage-display antibody library. Many of these antibodies showed broadly neutralizing ability.

Structural characterization of HCV envelope glycoproteins is challenging because of the difficulty in obtaining homogenous protein preparations [10,21-23]. Recently, the crystal structure of E2 core bound to neutralizing antibody AR3C has been crystalized [10], The antibody AR3C belongs to a group of broadly neutralizing antibodies that recognize antigenic region 3 (AR3) of E2 protein and cross-neutralizes HCV genotypes by blocking CD81 receptor binding site [14].

In this study, we characterized the B-cell epitope from the E2c-AR3C structure. By mapping this B-cell epitope to HCV E2 protein sequences, all strains available in the HCV database have been catalogued and compared with the known neutralized HCV strains. We examined the B-cell epitope diversity among the HCV variants, assessed potential cross-neutralization of the broadly neutralizing antibody across all sequences, and provided suggestions for selection of representative strains for future analysis of diversity and cross-recognition of HCV neutralizing B-cell epitopes.

## Materials and methods

### Structures of neutralizing antibody-E2 core protein complex

HCV envelope glycoproteins E1 and E2 mediate fusion and entry into host cells and are the primary targets of the humoral immune responses. The structure of the E2 core bound to a broadly neutralizing antibody was first crystalized at 2.65 angstroms [10], and deposited in PDB [19] database (PDB ID: 4MWF).

### Sequences of E2 protein from Hepatitis C virus

All E2 envelope protein sequences of HCV strains were retrieved from HCV database [24] (http://hcv.lanl.gov/content/index), a database that provides annotated data about HCV sequences. We retrieved 5589 E2 sequences from the HCV database. Of these, 5340 sequences with translated protein sequences were retained in E2 protein dataset, with 3723, 275, 995, 70, 22 and 87 sequences labeled as genotype 1-6, respectively. Among these, 168 sequences were genotype-unclassified isolates or representatives of recombinant strains. Five of the seven neutralizing motifs studied in [18] were represented in this E2 data set.

### Neutralizing activity of monoclonal antibody AR3C

The comparison of mAbs binding to the antigenic regions 1(AR1), 2(AR2), and 3(AR3) showed that 3(AR3)-specific mAbs reacted not only with genotype 1, but also genotype 2a, suggesting the presence of highly conserved epitopes in AR3 [18]. Table 1 shows the neutralizing activity data from this study. The mAb AR3C neutralized multiple genotypes: 1a, 1b, 2a, 2b, 4 and 5. We retrieved the E2 sequences of these isolates from GenBank [25].

**Table 1:** Discontinuous motifs on B-cell epitope and its surrounding area.

Isolate	Geno-type	IC_50 _(µg/ml)^a^	**Genbank accession no**.	Discontinuous motif	
				**B-cell epitope**	**Surrounding area**

4mwfC				ILNCN**E**SLGLALFY***K***CW	NTWGQSAY
H77	1a	1	AF009606.1	ILNCN**E**SLGLALFY***K***CW	NTWGQSAY
H		1	M67463.1	ILNCN**E**SLGLALFY***K***CW	NTWGQSAY
OH8	1b	5	AY545951.1	ILNCNDSLGLALFY***R***CW	QTFAANDY
UKN1 B12.16		1	AY734974.1	ILNCNDSLGLALFY***N***CW	NTFAVTEY
JFH-1	2a	1	AB047639.1	ILNCNDSLGLALFY***R***CW	NTFATTEY
J6		10	AF177036.1	ILNCNDSLG**I**ALFY***S***CW	HTFSTTEY
UKN2 A1.2		10	AY734977.1	ILNCNDSLG**I**ALFY***S***CW	QTFSTTEY
UNK2 B1.1	2b	10	AY734982.1	ILNCNDSLGLALFY***N***CW	QTFSVSEY
UKN4 21.16	4	1	AY734987.2	ILNCNDSLGLALFY***S***CW	NTFGHNEY
UKN5 15.7	5	1	EF427672.1	ILNC**Q**DSLG**I**A**L**LY***K***CW	QTFGFNSY

^a ^Neutralization activity of E2-specific neutralizing antibody AR3C (IC_50 _data are from reference [18]).

Comparing the discontinuous motifs on B-cell epitope, residues differing from the consensus are underlined, and the highly variable position lacking a consensus residue is underlined and in italics.

### Consistency of strain sequence numbering

All sequences in E2 protein dataset were aligned using MAFFT multiple alignment server [26]. The multiple sequence alignment (MSA) results provided a consistent sequence numbering scheme for further analysis of all sequences.

For each validated strain (Table 1), sequence similarity to all sequences in E2 protein dataset was assessed using BLAST [27] search. The sequence from E2 protein dataset with the highest identity score was used as the reference sequence. This step also provided a consistent sequence numbering scheme of positions within the MSA results for validated strains.

### Identification of B-cell epitope and surrounding area

Usintg crystal structures of the antigen-antibody complex, we defined antigen-binding sites (B-cell epitopes) as described previously [28,29]. This was done using both the measurements of residue Accessible Surface Area (ASA) and the minimum atom distance to the antibody.

a) For each residue on antigen protein, the ASA value was calculated using Naccess [28] software for free antigen and for antigen coupled with the corresponding antibody. Residues *r_i _*with ASA loss more than 20% were selected as designated epitope residues,

$$r_{i}\in \left\{epitope\;residues\right\}\;if\frac{ASA_{free}-ASA_{coupled}}{ASA_{free}}>0.2$$

b) The majority of contacts between two interacting atoms occur at <5Å separation. Euclidean distance was calculated between atom *a_i _*and *a_j _*with their coordinates *a_i_(x_i_, y_i_, z_i_) *and *a_j_(x_j_, y_j_, z_j_) *in the PDB structure data,

$$d_{ij}=\sqrt{{\left(x_{i}-x_{j}\right)}^{2}+{\left(y_{i}-y_{j}\right)}^{2}+{\left(z_{i}-z_{j}\right)}^{2}}$$

Antigen residues *r_i _*whose minimum atom distance to the binding antibody is less than 4Å were also incorporated in epitope. The least atom distance was defined as

$$d_{min}=min\left\{d_{ij}\right\},\;a_{i}\in antigen\;residue\;r_{i},a_{j}\in antibody\;residue\;r_{j}$$

$$r_{i}\in \left\{epitope\;residues\right\}\;if\;d_{min}<4Å$$

The residues that satisfy either of these two conditions (ASA loss or the minimum distance thresholds) were considered to constitute a B-cell epitope.

For the definition of surrounding area, we continued to use distance-based method: antigen residues with minimum atom distance to binding antibody less than 6Å, that are not B-cell epitope residues, were incorporated as components of the surrounding area.

### Extraction of discontinuous motifs (functional motifs)

**Figure 1:**
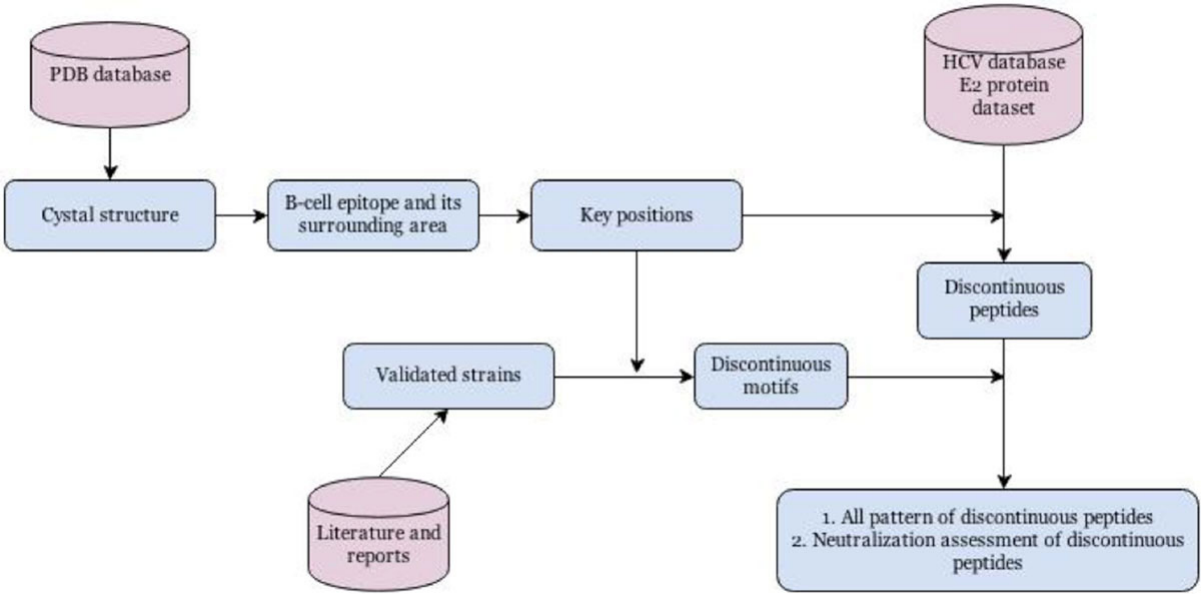
**The workflow used in this study**. The steps included: identification of a B-cell epitope and its surrounding area(key residues) from crystal structure, extraction of discontinuous motifs and peptides by key positions, cataloging and neutralization assessment of strains in E2 protein dataset by discontinuous peptides.

Based on the BLAST result, residue positions of a B-cell epitope and its surrounding area identified on the crystal structure were mapped onto its reference sequence, and further transferred to map onto all validated strain sequences (Figure 1). For structure sequence or each of the validated strain sequences, a residue string from these epitope positions was recognized as a discontinuous motif. Since we do not have negative data (escape variants), all discontinuous motifs extracted from these strains were classified as neutralized motifs, which were recognized as functional in neutralizing assays.

### Extraction of discontinuous peptides

The concept of discontinuous peptide [31] describes a virtual linear residue string generated from sequences that combines residues that form B-cell epitope that are not continuous in the protein sequence. Discontinuous peptides were extracted from the E2 protein dataset. Based on the BLAST and MSA results, the residue positions of B-cell epitope and its surrounding area were mapped onto its reference strain sequence, and then mapped onto all sequences in E2 protein dataset (Figure 1). Patterns of discontinuous peptides were used to catalog all strains in the dataset, and they were compared to the functional neutralized motifs. Each discontinuous peptide that has unique sequence was termed a discontinuous motif.

## Results

### Neutralizing antibody against HCV E2c protein

**Figure 2:**
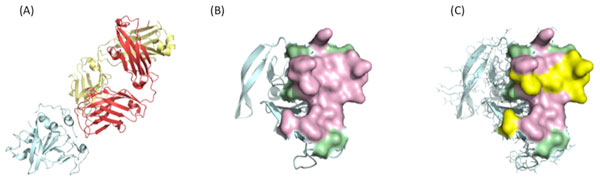
**The B-cell epitope and surrounding area recognized by neutralizing antibody AR3C**. (A) The heavy and light chains of mAb AR3C are shown in red and yellow respectively and the E2 chain in light blue; (B) The B-cell epitope on E2c is highlighted in pink (4MWF, chain C: 422, 427, 428, 429, 430, 431, 432, 433, 436, 438, 439, 441, 442, 443, 446, 503 and 529), and the ring area surrounding B-cell epitope is green (4MWF, chain C: 434, 435, 437, 440, 444, 528, 531 and 613); (C) The variable residues which are different from mAb AR3C-neutralized are highlighted in yellow (4MWF, chain C: 422, 430, 431, 432, 433, 438 and 442).

The mAb AR3C was known to neutralize HCV genotype 1, 2, 4 and 5. We performed the analysis of the structure of mAb AR3C complexed with HCV E2c. The B-cell epitope and its surrounding area in structure 4MWF were identified (Figure 2) as described in the MATERIAL AND METHODS section.

### Functional motifs on B-cell epitopes and its surrounding area

The positions of B-cell epitope residues were extracted and mapped to all validated strain sequences. Functional motifs were retrieved with corresponding neutralizing information. Seven distinct discontinuous motifs (identical motifs were present across different strains) were extracted from the sequences of E2 protein structure and 10 validated strains.

### Discontinuous peptides derived from B-cell epitopes

**Figure 3:**
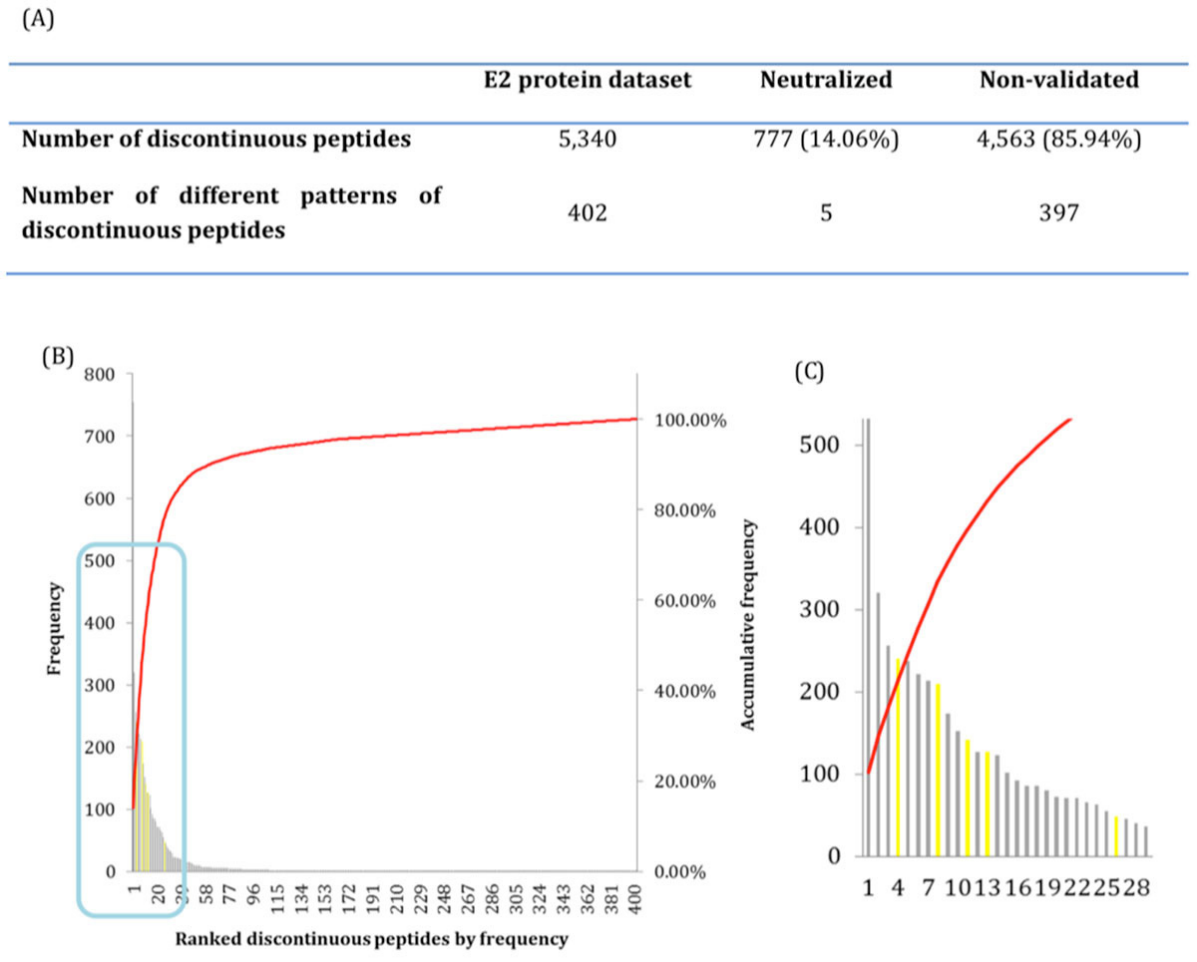
**An overview of discontinuous peptides in the E2 protein dataset**. (A) The number of discontinuous peptides and the number of discontinuous motifs generated from E2 protein dataset; (B) The distribution of all discontinuous peptide patterns frequencies. The yellow and grey bars represent discontinuous peptides identical to the neutralized motifs and the ones without validation data yet, while the red line is their accumulative frequency; (C) The zoom-up view of top ranked discontinuous peptides frequencies, from (B).

The positions of epitope residues were mapped onto all sequences in the E2 protein dataset. Amino acid string representing discontinuous peptide was extracted from each E2 protein sequence. Among all 5340 sequences in E2 protein dataset, there were 402 different combinations of discontinuous peptides (patterns), which reflect the high sequential variability of HCV virus. Five discontinuous peptides identical to discontinuous motifs from neutralized strains covered 14.06% strains population (Figure 3A). The discontinuous peptides were further sorted according to their frequencies in the E2 protein dataset. Viewed by ranked frequencies, the top 10 most frequent discontinuous peptides covered more than 50% strains in the dataset, and top 25 discontinuous peptides covered nearly 80% of the total strain population (Figures 3B and 3C).

Top ranked discontinuous peptides and those identical to the discontinuous motifs extracted from the E2 protein dataset are listed in Table 2 along with their frequencies. The most frequent discontinuous peptide has coverage of 754 strains, while the second most frequent peptide covers 320 strains. There is no validation data for the 3 most frequent discontinuous peptides, while discontinuous motifs ranked 4^th^, 6^th^, 11^th^, 12^th^, and 26^th ^in the list were shown to be neutralizing. The neutralization potential of these un-tested discontinuous motifs could be estimated by comparing the composing amino acids to the validated motifs. The 1^st ^ranked discontinuous peptide (**ILNCNDSLGIALFYKCW**) is different from the 4^th ^ranked discontinuous peptide (**ILNCNDSLGLALFYRCW**, which is a neutralized motifs) in two positions: 10^th ^residue L->I, and 15^th ^residue R->K. Since both residues share similar chemical features, it is possible that the HCV strains with 1^st ^discontinuous peptides could be neutralized by the mAb AR3C. Also, the two different residues have been shown in other validated neutralized motifs: the 26^th ^ranked (**ILNCNDSLGIALFYSCW**) and 6^th ^ranked (**ILNCNESLGLALFYKCW**) discontinuous peptides. From the reported neutralizing data, we derived the consensus sequence for B-cell epitope ILNCNDSLGIALFYKCW and experimentally verified E2 neutralizing motif I-L-N-C-[NQ]-[DE]-S-L-G-[IL]-A-L-F-Y-[KNRS]-C-W. Potentially neutralizing motif that should be validated experimentally is [IVL]-L-[NS]-C-[NQ]-[DEA]-[ST]-[LI]-G-[ILVM]-[ATV]-L-[FILM]-Y-X-[WF] (see Additional file 1). Targeted experimentation will identify B-cell epitope changes that would abolish AR3C binding as well as changes that do not have detrimental effects.

**Table 2:** Top ranked discontinuous peptides in the E2 protein dataset.

Rank	Discontinuous peptides	Frequency	Accumulative percentage	Validation status
1	ILNCNDSLGIALFYKCW	754	14.12%	Missing
2	ILNCNDSLGLALFYKCW	320	20.11%	Missing
3	ILNCN***A***SLGIALFYKCW	256	24.91%	Missing
4	ILNCNDSLGLALFYRCW	**240**	**29.40%**	**Neutralized**
5	ILNCN***A***SLG***V***ALFYKCW	237	33.84%	Missing
6	ILNCNESLGLALFYKCW	**221**	**37.98%**	**Neutralized**
7	ILNCNDSLGIAL***I***YKCW	213	41.97%	Missing
8	ILNCN***A***SLGLALFYRCW	209	45.88%	Missing
9	ILNCNES***I***GIALFYKCW	173	49.12%	Missing
10	ILNCNDSLGIALFYRCW	152	51.97%	Missing
11	ILNCNDSLGLALFYNCW	**141**	**54.61%**	**Neutralized**
12	ILNCNDSLGLALFYSCW	**127**	**56.99%**	**Neutralized**
13	ILNCND***TI***GIALFYRCW	127	59.36%	Missing
14	ILNCNDS***I***GIALFYRCW	123	61.67%	Missing
15	ILNCNDSLGIAL***L***YKCW	102	63.58%	Missing
16	***V***LNCNES***I***GLALFYKCW	92	65.30%	Missing
17	ILNCNDSLG***V***ALFYKCW	85	66.89%	Missing
18	ILNCN***A***SLGLALFYKCW	85	68.48%	Missing
19	ILNCN***A***SLG***V***AL***L***YKCW	80	69.98%	Missing
20	ILNCNDSLGIALFYNCW	72	71.33%	Missing
21	ILNC***D***ES***I***GIALFYKCW	71	72.66%	Missing
22	ILNCNDS***I***GIALFYKCW	71	73.99%	Missing
23	ILNCNES***I***GLALFYKCW	66	75.22%	Missing
24	ILNCNDSLG***V***AL***L***YKCW	63	76.40%	Missing
25	***L***LNCNDSLGLALFYKCW	55	77.43%	Missing
26	ILNCNDSLGIALFYSCW	**48**	**78.33%**	**Neutralized**

The top 26 most frequent (including discontinuous peptides identical to discontinuous neutralized motifs) among 402 different patterns of discontinuous peptides are listed. This table lists discontinuous peptide, their frequencies, accumulative frequency and neutralization validation status to mAb AR3C. The neutralized discontinuous motifs are underlined. The residues in italics and underlined, indicate the amino acids that are not presented in the specific position from these known neutralized discontinuous motifs (see Figure 1(c)).

**Figure 4:**
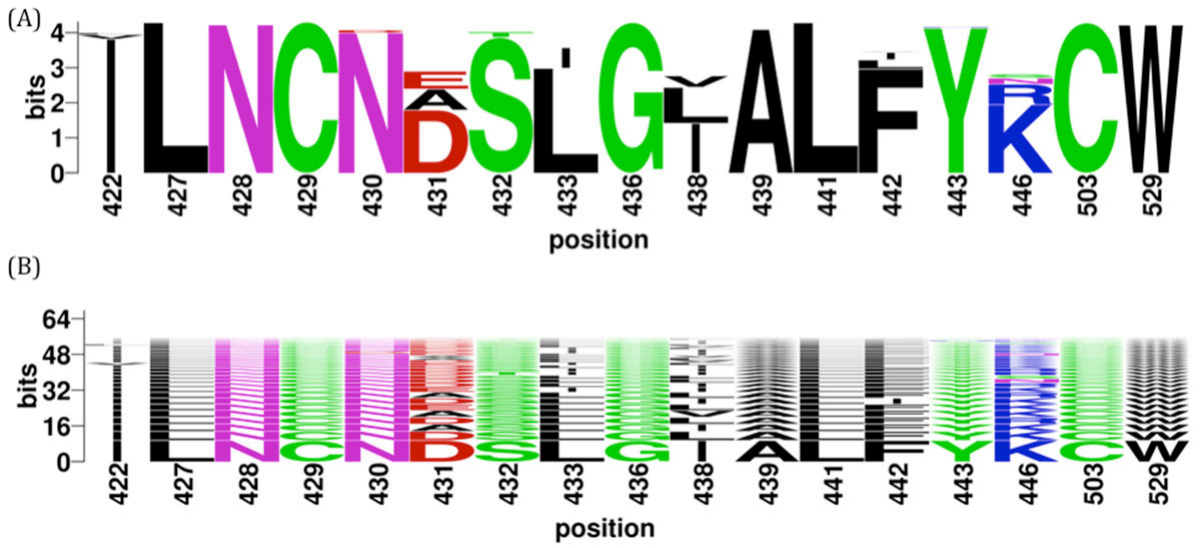
**Discontinuous peptides generated from the B-cell epitope of mAb AR3C**. Discontinuous peptides were extracted from 5340 E2 protein sequences according to relevant positions from the PDB structure 4MWF, chain C: 422, 427, 428, 429, 430, 431, 432, 433, 436, 438, 439, 441, 442, 443, 446, 503 and 529. The corresponding positions in the reference strain sequence (Genbank accession number ACA53572.1) are identical to the PDB structure. (A) WebLogo figure [29,34] and (B) BlockLogo figure [30] of discontinuous peptides from the E2 protein dataset.

WebLogo [29] and BlockLogo [30] were generated for all the discontinuous peptides extracted from E2 protein dataset. Among the 17-residue B-cell epitope, most of the positions are quite conserved, as shown in WebLogo figure (Figure 4A). However, the BlockLogo figure shows a large number of different combinations and the high diversity of this binding site (Figure 4B).

**Figure 5:**
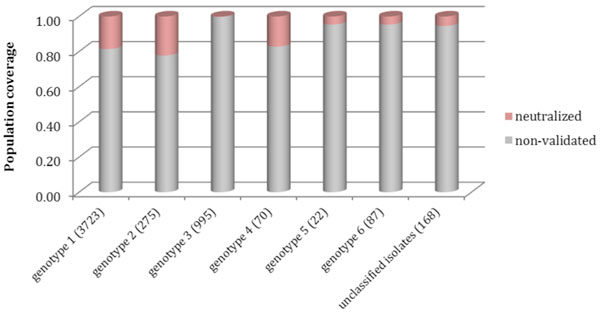
**MAb AR3C neutralization assessment sorted by HCV genotype**. The virus population coverage for each genotype and unclassified isolates, the number in bracket after each genotype indicate the number of strain sequences for specific genotype (based on data from E2 protein dataset). The proportion of discontinuous peptides identical to neutralized motifs is colored in pink, while non-validated in grey. For each genotype from left to right, the numbers of different motif patterns among these sequences are 280, 51, 70, 25, 12, 45 and 30.

The neutralized motifs cover 14.06% of strain sequences in the E2 protein dataset, while the other discontinuous peptides that cover 85.94% of the strains lack validated data (Figure 5). Viewed by the genotype, the neutralizing coverage of genotypes 1, 2 and 4 are approximately 20% (18.48%, 22.18% and 17.14% respectively), higher than those of genotype 3, 5 and 6. The overall known neutralized coverage on the dataset is low. Of 402 discontinuous peptides, 379 had a complete B-cell epitope and 15 had ambiguities in sequence (residue X). Eight sequences had disrupted B-cell epitope (patterns 38, 65, 93, 180, 214, 285, 385, and 387, Additional file 2) most likely representing non-viable viruses.

### Discontinuous peptides on B-cell epitope surrounding area

**Figure 6:**
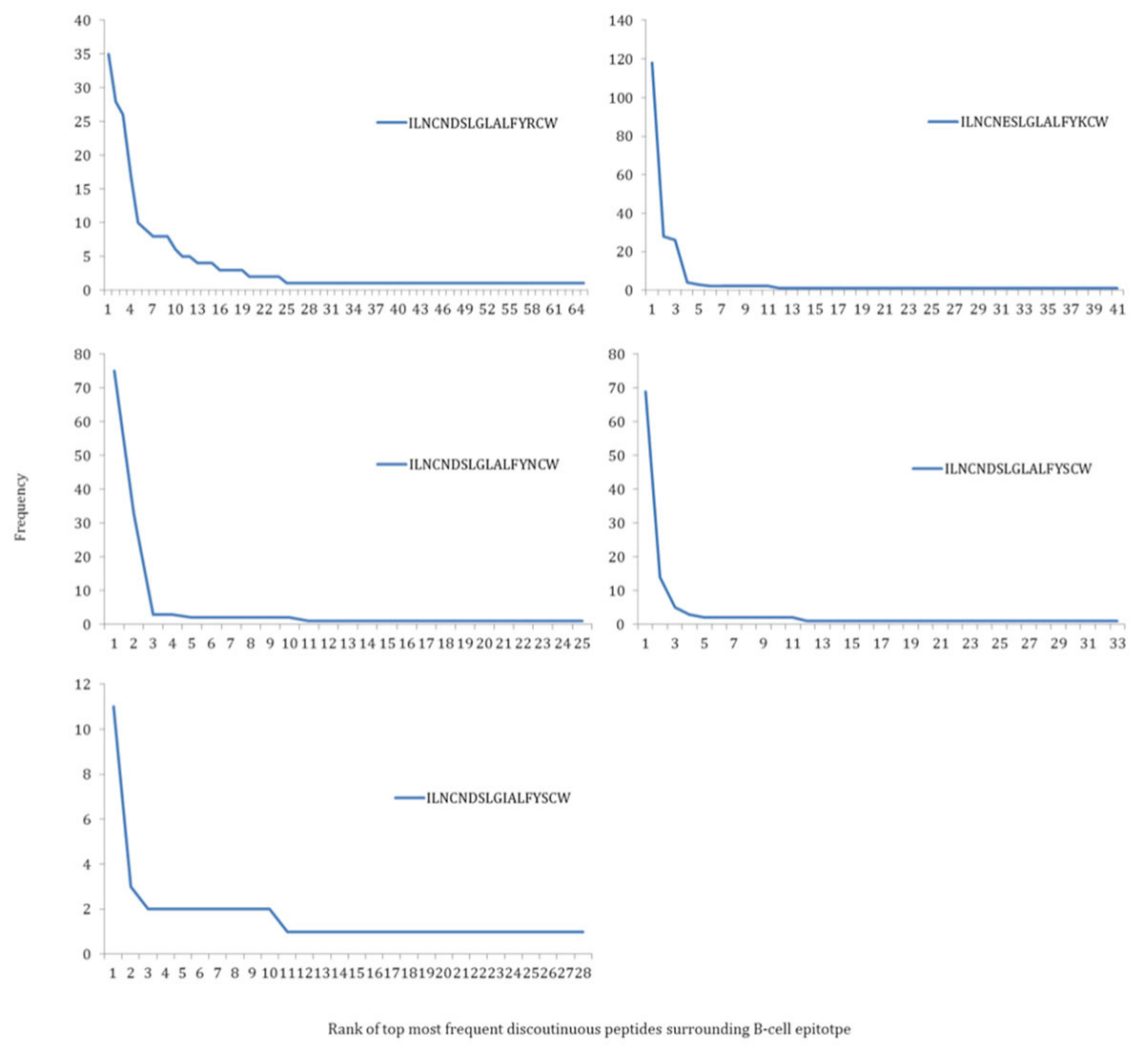
**Distribution of discontinuous peptides frequency on surrounding area of strains with discontinuous peptides identical to neutralized motifs on B-cell epitope**. (A) - (E), corresponds to five groups of strains share same discontinuous peptides on B-cell epitope identical to five neutralized motifs. The discontinuous peptides on B-cell epitope (neutralized motifs) of each group are listed on each plot.

The antibody binding and neutralization ability can possibly be affected by the B-cell epitope surrounding area. Identical discontinuous peptides on B-cell epitope alone cannot fully guarantee the same neutralization result. The analysis of surrounding area aims to provide a more detailed assessment about the potential neutralizing properties of the AR3C. The frequency distribution of different discontinuous peptides on surrounding area showed similarity to the results of B-cell epitope comparisons (Figure 6). For the strains share identical discontinuous peptides on B-cell epitope, the discontinuous peptides on surrounding area have dominant patterns: the top 5 patterns cover as much as 50% of the strains. The result indicates that the residues that define AR3C epitope surrounding area do not affect B-cell epitope/antibody interaction independently of the actual B-cell epitope.

## Conclusions and discussion

Hepatitis C virus, with its extreme variability of sequence repertoire, is a difficult target for vaccine design. Compared to envelope glycoproteins in other virus, such as hemagglutinin protein from influenza virus and E protein from dengue (DENV) virus, the B-cell epitopes on HCV E2 protein are much less conserved in composing residues. The MAb F10 [31] is a broadly neutralizing antibody against HA protein of influenza A virus. A total of 589 different patterns of discontinuous peptides on F10 B-cell epitope were extracted from 45,812 HA sequences. The mAb 2H12 [32] is a broadly neutralizing antibody shown to neutralize serotypes DENV1, 3 and 4, has 57 different patterns of discontinuous peptides on B-cell epitope that cover 4,659 dengue E protein sequences in dengue dataset [33]. In the current study, 5340 E protein sequences from HCV, which is a similar sequence set size as in dengue viruses, generated almost an order of magnitude larger diversity: 402 different discontinuous peptides at the mAb AR3C binding site have been identified.

We assembled a HCV strains cataloguing method in this study. Strains with identical discontinuous peptides on B-cell epitope site were grouped and estimated to have similar neutralizing activity. For mAb AR3C, the discontinuous peptides on B-cell epitope from validated strains ranked 4^th^, 6^th^, 11^th^, 12^th ^and 26^th^, covered 14.06% of all 5,340 strains in the E2 protein dataset. The discontinuous peptide and frequency list could be used as guidance for the selection of representative strains for future systematic neutralizing antibody tests. For example, the most dominant discontinuous peptides among population should be tested for neutralization assay in priority. For mAbs generated in the future, the neutralization coverage among the strains with top dominant discontinuous peptide could be used as a guidance of how broadly neutralized the mAb could reach.

The neutralizing motif indicates that conservative replacements at positions 430 (N→Q), 431 (D→E) and 438 (L→I) will likely not affect binding affinities sufficiently to abolish neutralization. In addition, position 446 has multiple residues observed in neutralized variants (K,N,S,R) and it appears not to affect antibody binding. By observation of common discontinuous peptides we argue that conserved positions 427 (L), 428 (N), 429 (C), 436 (G), 439 (A), 441 (L), 443 (Y), 503 (C), and 529 (W) have structural or functional significance. The positions 422, 431, 432, 433, 438, and 442 are key for the study of the diversity of B-cell epitopes and targeting the design of broadly-protective vaccines.

This results presented here are based on the existing data. More comprehensive conclusions will be generated as additional neutralizing antibody structures are crystallized and more neutralization assays are performed in the future. Advances in computation and biotechnology enable more comprehensive analysis where all combinations of antibodies and antigens can be assessed *in silico*. The new methodology of Big Data analysis [35] enables the analysis of diverse data types where protein, nucleotide, structure, and functional data can be analyzed in combination. The well-annotated data are combined with specialized analytical tools, including statistical analyses, sequence analysis, and mathematical models to gain insights into biological processes, generate knowledge, and inform decisions about validation experiments. This study has shown that the majority of common HCV variants have not been studied in antibody neutralization studies. The knowledge of cross-neutralization is, therefore, incomplete and there is an urgent need for designing libraries of viruses that will be representative of the majority of HCV strains. These libraries will enable systematic testing of strains against the panels of antibodies and enable the design of universal broadly protective HCV vaccines.

## Competing interests

The authors declare that they have no competing interests.

## Authors' contributions

VB and JS designed the study. JS collected HCV data from public database and performed the analysis. VB and JS draft the manuscript.

## Supplementary Material

Additional file 1 **Potentially neutralizing motifs derived from full list of discontinuous peptides in the E2 protein dataset**. (*.pdf). Click here for file

Additional file 2 **Full list of ranked discontinuous peptides in the E2 protein dataset**. (*.xls). Click here for file

## Declaration

Publication charges for this article have been funded by Nazarbayev University.

This article has been published as part of *BMC Medical Genomics *Volume 8 Supplement 4, 2015: Joint 26th Genome Informatics Workshop and 14th International Conference on Bioinformatics: Medical genomics. The full contents of the supplement are available online at http://www.biomedcentral.com/bmcmedgenomics/supplements/8/S4.
