# Supplementary material for: A systematic analysis of a broadly neutralizing antibody AR3C epitopes on Hepatitis C virus E2 envelope glycoprotein and their cross-reactivity
Source: BMC Med Genomics. 2015 Dec 9;8(Suppl 4):S6. doi: 10.1186/1755-8794-8-S4-S6 (PMC4682370; doi:10.1186/1755-8794-8-S4-S6)
Supplement: Additional file 1 — Potentially neutralizing motifs derived from full list of discontinuous peptides in the E2 protein dataset. (*.pdf). [file 1755-8794-8-S4-S6-S1.pdf]

A large-scale analysis of a broadly neutralizing antibody AR3C epitopes on Hepatitis C virus E2 envelope glycoprotein and their cross-reactivity

Jing Sun[1], Vladimir Brusic[2]

jing_sun@dfci.harvard.edu

vladimir.brusic@nu.edu.kz

[1]Dana-Farber Cancer Institute, Harvard Medical School, Boston, MA, USA

[2] School of Science and Technology, Nazarbayev University, Astana, Kazakhstan

Supplementary material

Table S1. Full list of ranked discontinuous peptides in the E2 protein dataset. 402 different patterns of discontinuous peptides are listed. This table lists discontinuous peptide, their frequencies, accumulative frequency and validation status.

Rank	Discontinuous peptides	Frequency	Accumulative percentage	Validation status
1	ILNCNDSLGIALFYKCW	754	14.12%	Missing
2	ILNCNDSLGLALFYKCW	320	20.11%	Missing
3	ILNCNASLGIALFYKCW	256	24.91%	Missing
**4**	**ILNCNDSLGLALFYRCW**	**240**	**29.40%**	**Neutralized**
5	ILNCNASLGVALFYKCW	237	33.84%	Missing
**6**	**ILNCNESLGLALFYKCW**	**221**	**37.98%**	**Neutralized**
7	ILNCNDSLGIALIYKCW	213	41.97%	Missing
8	ILNCNASLGLALFYRCW	209	45.88%	Missing
9	ILNCNESIGIALFYKCW	173	49.12%	Missing
10	ILNCNDSLGIALFYRCW	152	51.97%	Missing
**11**	**ILNCNDSLGLALFYNCW**	**141**	**54.61%**	**Neutralized**
**12**	**ILNCNDSLGLALFYSCW**	**127**	**56.99%**	**Neutralized**
13	ILNCNDTIGIALFYRCW	127	59.36%	Missing
14	ILNCNDSIGIALFYRCW	123	61.67%	Missing
15	ILNCNDSLGIALLYKCW	102	63.58%	Missing
16	VLNCNESIGLALFYKCW	92	65.30%	Missing
17	ILNCNDSLGVALFYKCW	85	66.89%	Missing
18	ILNCNASLGLALFYKCW	85	68.48%	Missing
19	ILNCNASLGVALLYKCW	80	69.98%	Missing

20	ILNCNDSLGIALFYNCW	72	71.33%	Missing
21	ILNCDESIGIALFYKCW	71	72.66%	Missing
22	ILNCNDSIGIALFYKCW	71	73.99%	Missing
23	ILNCNESIGLALFYKCW	66	75.22%	Missing
24	ILNCNDSLGVALLYKCW	63	76.40%	Missing
25	LLNCNDSLGLALFYKCW	55	77.43%	Missing
**26**	**ILNCNDSLGIALFYSCW**	**48**	**78.33%**	**Neutralized**
27	ILNCNESLGIALFYKCW	46	79.19%	Missing
28	ILNCNDSLGIALFHKCW	40	79.94%	Missing
29	ILNCNDTLGLALFYKCW	36	80.62%	Missing
30	ILNCNASLGLALFYNCW	34	81.25%	Missing
31	ILNCNESLGLALVYKCW	32	81.85%	Missing
32	ILNCNASLGIALLYKCW	29	82.40%	Missing
33	ILNCNDSIGIALIYKCW	23	82.83%	Missing
34	ILNCNASLGVALFYRCW	23	83.26%	Missing
35	ILNCDDSLGIALFYKCW	23	83.69%	Missing
36	ILNCNASLGIALFYQCW	22	84.10%	Missing
37	ILNCNESLGIALFYRCW	22	84.51%	Missing
38	--------------E--	20	84.89%	Missing
39	ILNCNESLGLALIYKCW	20	85.26%	Missing
40	ILNCNDSLGLVLFYNCW	18	85.60%	Missing
41	ILNCNESIGIALFYRCW	17	85.92%	Missing
42	ILNCNESLGLALLYKCW	17	86.24%	Missing
43	ILNCNDSLGIALLYRCW	16	86.54%	Missing
44	VLNCNDSLGLALFYKCW	15	86.82%	Missing
45	ILNCNESLGLALFYRCW	15	87.10%	Missing
46	ILNCNESLGVALFYKCW	15	87.38%	Missing
47	ILNCNDSLGVALFYRCW	13	87.62%	Missing
48	ILNCNDSLGFALFYKCW	12	87.85%	Missing
49	ILNCNESLGVALLYKCW	11	88.05%	Missing
50	ILNCNDSIGLALFYKCW	10	88.24%	Missing
51	ILNCNDSLGIALMYKCW	9	88.41%	Missing
52	ILNCNDTLGIALLYKCW	9	88.58%	Missing
53	ILNCNASLGIALIYKCW	9	88.75%	Missing
54	ILNCNDTLGIALFYKCW	9	88.91%	Missing
55	ILNCNDSLGIALIYRCW	8	89.06%	Missing
56	VLNCNESIGLALFYECW	7	89.19%	Missing
57	ILNCNASLGVALLYSCW	7	89.33%	Missing
58	ILNCNDSLGIALLYNCW	7	89.46%	Missing
59	ILNCNDSLGMALFYKCW	7	89.59%	Missing
60	ILNCNESLGLALFYNCW	7	89.72%	Missing
61	ILNCNDTLGLALFYRCW	7	89.85%	Missing
62	ILNCNDSIGFALFYKCW	7	89.98%	Missing

63	ILNCNDSLGVALFYNCW	7	90.11%	Missing
64	ILNCNASIGVALLYKCW	6	90.22%	Missing
65	LLNCTE--------PCW	6	90.34%	Missing
66	VLNCNDSLGIALFYRCW	6	90.45%	Missing
67	ILNCDDSLGLALFYKCW	5	90.54%	Missing
68	ILSCNDSLGLALFYKCW	5	90.64%	Missing
69	ILNCNESIGLALFYRCW	5	90.73%	Missing
70	ILNCNDSLGLALFYXCW	5	90.82%	Missing
71	ILNCNDTLGIALFYRCW	5	90.92%	Missing
72	ILNCNDSLGIALFYTCW	5	91.01%	Missing
73	ILNCNDSLGIALFYHCW	5	91.10%	Missing
74	ILNCNDSLGISLFYKCW	5	91.20%	Missing
75	ILNCNESIGVALFYKCW	5	91.29%	Missing
76	ILNCNDSLGIALVYKCW	5	91.39%	Missing
77	ILNCNDSLGLALFYGCW	5	91.48%	Missing
78	ILNCNDSLGLALLYKCW	4	91.55%	Missing
79	VLNCNDSLGLALFYRCW	4	91.63%	Missing
80	ILNCNDSLGITLFYKCW	4	91.70%	Missing
81	ILNCNESLGVALIYKCW	4	91.78%	Missing
82	ILNCNDSLGLALIYKCW	4	91.85%	Missing
83	ILNCNDSIGFALFYSCW	4	91.93%	Missing
84	VLNCNDSLGLALFYKCW	4	92.00%	Missing
85	ILNCNASLGVALIYKCW	4	92.08%	Missing
86	ILNCKDSLGIALFYKCW	4	92.15%	Missing
87	ILNCNASHGVALLYSCW	4	92.23%	Missing
88	ILNCNDSLGMALFYRCW	3	92.28%	Missing
89	ILNCNDSLGITLFYSCW	3	92.34%	Missing
90	ILNCNESLGIALLYKCW	3	92.40%	Missing
91	ILNCNDSLGITLFYRCW	3	92.45%	Missing
92	ILNCNASIGIALLYKCW	3	92.51%	Missing
93	-----------------	3	92.57%	Missing
94	ILNCNASLGLALFYQCW	3	92.62%	Missing
95	ILNCNASLGITLFYKCW	3	92.68%	Missing
96	ILNCNASLGVALIYRCW	3	92.73%	Missing
97	ILSCNDSLGMALFYKCW	3	92.79%	Missing
98	ILNCNTSLGLALFYKCW	3	92.85%	Missing
99	ILNCNDSLGLSLFYKCW	3	92.90%	Missing
100	ILNCNDSLGFALFYRCW	3	92.96%	Missing
101	ILNCNASLGIALFYRCW	3	93.01%	Missing
102	VLNCNESLGLALFYKCW	3	93.07%	Missing
103	LLNCFDSLGLALFAKCS	3	93.13%	Missing
104	ILNCNSSLGIALFYKCW	3	93.18%	Missing
105	ILNCNESLGMALFYKCW	3	93.24%	Missing

106	ILNCNESIGFALFYKCW	3	93.30%	Missing
107	ILNCNDSLGISLFYRCW	3	93.35%	Missing
108	VLNCNTSLGLALFYKCW	3	93.41%	Missing
109	ILNCNDSLGLTLFYKCW	3	93.46%	Missing
110	ILNCNSSLGLALFYKCW	2	93.50%	Missing
111	ILNCNDTLGIALFYHCW	2	93.54%	Missing
112	VLSCNDSLGIALFYRCW	2	93.58%	Missing
113	ILNCNESIGIALLYKCW	2	93.61%	Missing
114	ILNCNESIGIALIYKCW	2	93.65%	Missing
115	ILNCNASHGVALFYRCW	2	93.69%	Missing
116	ILNCNDSLGVALIYKCW	2	93.73%	Missing
117	ILSCNDSLGVALFYRCW	2	93.76%	Missing
118	ILNCNASLGLALLYKCW	2	93.80%	Missing
119	ILNCNASIGIALIYSCW	2	93.84%	Missing
120	ILDCDDSLGIALFYKCW	2	93.88%	Missing
121	ILNCNDTLGIALFYSCW	2	93.91%	Missing
122	ILNCNDTLGLVLFYNCW	2	93.95%	Missing
123	ILNCNENIGIALFYQCW	2	93.99%	Missing
124	ILNCNDSLGLALVYRCW	2	94.03%	Missing
125	VLNCNDSLGIALMYKCW	2	94.06%	Missing
126	ILNCNASLGIALFYNCW	2	94.10%	Missing
127	ILNCNEGLGVALFYKCW	2	94.14%	Missing
128	ILNCNDSLGLALLYRCW	2	94.18%	Missing
129	ILNCNASLGLALFSKCW	2	94.21%	Missing
130	ILNCNASLGVALFYSCW	2	94.25%	Missing
131	ILNCYDSLGVALFYKCW	2	94.29%	Missing
132	ILNCNDSLGIALVYRCW	2	94.33%	Missing
133	ILNCNDPIGIALIYKCW	2	94.36%	Missing
134	ILNCNASLGIALFHKCW	2	94.40%	Missing
135	ILNCNASIGIALMYSCW	2	94.44%	Missing
136	ILNCNDTLRPALFYKCW	2	94.48%	Missing
137	ILNCNDSLGXALFYKCW	2	94.51%	Missing
138	ILNCNDSLGLALFYHCW	2	94.55%	Missing
139	ILNCNDSLGVALIYRCW	2	94.59%	Missing
140	ILNCNDSLGFTLFYKCW	2	94.63%	Missing
141	ILNCNASHGVALFYKCW	2	94.66%	Missing
142	ILNCDDSIGIALFYKCW	2	94.70%	Missing
143	ILNCNESIGIALMYKCW	2	94.74%	Missing
144	LPNCNDSLGLALFYKCW	2	94.78%	Missing
145	ILNCIDSLGLSLFYNCW	2	94.81%	Missing
146	ILNCNDSHGLALFYKCW	2	94.85%	Missing
147	VLNCNESLGIALFYSCW	2	94.89%	Missing
148	ILNCNDSLGVALFYSCW	2	94.93%	Missing

149	ILNCNASLGLALFYSCW	2	94.96%	Missing
150	ILTCNGSLGIVLFYKCW	2	95.00%	Missing
151	ILSCNASLGLALFYKCW	2	95.04%	Missing
152	ILNCNESLGLALFYSCW	2	95.07%	Missing
153	ILNCNDSLGVALLYRCW	2	95.11%	Missing
154	ILNCNSSLGVALLYKCW	2	95.15%	Missing
155	ILNCNVSIGVALLYKCW	2	95.19%	Missing
156	ILNCNASLGVALFYQCW	2	95.22%	Missing
157	ILNCNDSLGIALMYKCF	2	95.26%	Missing
158	ILNCNESLGFALFYKCW	2	95.30%	Missing
159	ILNCNESLGIALFCRCW	2	95.34%	Missing
160	ILNCNDSLGIALIYKCF	2	95.37%	Missing
161	ILNCNASLGVALFYNCW	2	95.41%	Missing
162	ILNCNASIGIALFYKCW	2	95.45%	Missing
163	ILNCNASLGMALFYNCW	2	95.49%	Missing
164	ILNCNDSLGLALFYTCW	2	95.52%	Missing
165	ILNCNDSLGIALFYXCW	2	95.56%	Missing
166	LLNCNDSLGIALFYRCF	1	95.58%	Missing
167	ILNCNSSLGVALFYKCW	1	95.60%	Missing
168	LLNCNDSLGLALLYKCW	1	95.62%	Missing
169	XLNCNDSLGIALFYRCW	1	95.64%	Missing
170	ILNCNASLGIALFYECW	1	95.66%	Missing
171	ILNCEASLGVALIYKCW	1	95.67%	Missing
172	ILNCNNSLGLALFYKCW	1	95.69%	Missing
173	ILNCDESIGMALFYKCW	1	95.71%	Missing
174	ILNCNASLGIALFHRCW	1	95.73%	Missing
175	ILNCNDSLGLALFYQCW	1	95.75%	Missing
176	ILNCNDSLGIALFYNCC	1	95.77%	Missing
177	ILNCNDSLGIHLFYTCW	1	95.79%	Missing
178	ILSCNDSLGLALFYRCW	1	95.81%	Missing
179	ILNCNDSLGFALFYSCW	1	95.82%	Missing
180	--------------G--	1	95.84%	Missing
181	ILNCXDSLGLALFYRCW	1	95.86%	Missing
182	ILNCNESLGLAXFYKXW	1	95.88%	Missing
183	VLNCNDSLGIALFYSCW	1	95.90%	Missing
184	ILNCDESLGLALLYKCW	1	95.92%	Missing
185	ILNCNASHGLALFYKCW	1	95.94%	Missing
186	ILNCNDSLGFALFYECW	1	95.96%	Missing
187	ILNCNDSLGLALFYNCF	1	95.97%	Missing
188	LLNCNDSLGLALFYKCX	1	95.99%	Missing
189	ILNCNASTGVALFYKCW	1	96.01%	Missing
190	ILNCNDSLGIALXYXCW	1	96.03%	Missing
191	IGNGNESNRLALIYKCW	1	96.05%	Missing

192	ILNCNTSLGLALFHKCW	1	96.07%	Missing
193	ILNCSDSLGVALFYNCW	1	96.09%	Missing
194	ILNCNDSXGFALFYKCW	1	96.10%	Missing
195	ILNCNDSLGIVLFYRCW	1	96.12%	Missing
196	ILNCNESIGVALFYRCF	1	96.14%	Missing
197	VLNCNGTLGLALFYKCW	1	96.16%	Missing
198	ILNCAKLLGVCLFYSCW	1	96.18%	Missing
199	ILNCNDSLGITLFYNCW	1	96.20%	Missing
200	ILNCHDSLGITLFYHCW	1	96.22%	Missing
201	ILNCNESLGLVLFYKCW	1	96.24%	Missing
202	ILNCNDSLGLTLFYQCW	1	96.25%	Missing
203	ILNCNASIGITLMYSCW	1	96.27%	Missing
204	VLNCNDSLGIALFYGCW	1	96.29%	Missing
205	ILNCNESLGIALIYKCW	1	96.31%	Missing
206	ILNCDESLGLALFYKCW	1	96.33%	Missing
207	ILNCNDTLGFALFYKCW	1	96.35%	Missing
208	LLNCNDSLGLALFHKCW	1	96.37%	Missing
209	TLNCNESIGIALFYKCW	1	96.39%	Missing
210	ILSCNDSLGLVLFYNCW	1	96.40%	Missing
211	ILNCNGSLGIVLFYKCW	1	96.42%	Missing
212	ILNCNDSLGVALFYKCF	1	96.44%	Missing
213	ILNCNDTLGIKLFYNCW	1	96.46%	Missing
214	AIDKYDLS------EVL	1	96.48%	Missing
215	ILNCNESPGIALFYKCW	1	96.50%	Missing
216	ILNCNDSLGISLFYSCW	1	96.52%	Missing
217	ILNCNESLGLTLFSHCW	1	96.54%	Missing
218	ILNCNDSLGLALFFKCW	1	96.55%	Missing
219	ILNCDASLGLALFYKCW	1	96.57%	Missing
220	ILSCNDSLGVALFYSCW	1	96.59%	Missing
221	ILNCHDSLGIALFYNCW	1	96.61%	Missing
222	ILNCNDTLGIALLYKCF	1	96.63%	Missing
223	ILNCNDSLGIALIYSCW	1	96.65%	Missing
224	LLNCNDSLGLALFYKXW	1	96.67%	Missing
225	ILNCNDTFGLALFYRCW	1	96.69%	Missing
226	ILNCNDSLGIALIYECW	1	96.70%	Missing
227	ILNCNDSLGLAPFYRCW	1	96.72%	Missing
228	ILNCNDSLGIALFAKCW	1	96.74%	Missing
229	ILNCNASLGVALLYNCW	1	96.76%	Missing
230	ILNCNESIGIALFYKRW	1	96.78%	Missing
231	ILNCNDSLGIALLYQCW	1	96.80%	Missing
232	ILSCNDSIGIALVYRCW	1	96.82%	Missing
233	ILNCNESLGLALLYRCW	1	96.84%	Missing
234	VLNCNDSLGLALFYNCW	1	96.85%	Missing

235	ILSCNASLGLTLFYKCW	1	96.87%	Missing
236	ILNCNDSLGLALFYECW	1	96.89%	Missing
237	ILTCNASLGLALFYKCW	1	96.91%	Missing
238	ILNCDESIGVALFYKCW	1	96.93%	Missing
239	ILNCNESLGIAPFYRCW	1	96.95%	Missing
240	ILNCNDSIGIALLYKCW	1	96.97%	Missing
241	VLNCDDSLGIALFYKCW	1	96.99%	Missing
242	ILNCNDSLGAALFYSCW	1	97.00%	Missing
243	ILNCNDSLGMALFYNCW	1	97.02%	Missing
244	ILNCKDSLGLALFYRCW	1	97.04%	Missing
245	ILNCNDSLGVTLFYKCW	1	97.06%	Missing
246	ILNCNDNLGMALLYKCW	1	97.08%	Missing
247	ILNCGESIGIALFYKCW	1	97.10%	Missing
248	LLNCNDSLGLALFYKRW	1	97.12%	Missing
249	ILTCNDSLGIALFYRCW	1	97.13%	Missing
250	ILNCNANLGLALFYKCW	1	97.15%	Missing
251	ILNCNDSIGVALLYKCW	1	97.17%	Missing
252	ILNCEKLLGVTLFYSCW	1	97.19%	Missing
253	VLNCNASLGVALLYRCW	1	97.21%	Missing
254	ILNCNASIGVALFYKCW	1	97.23%	Missing
255	VLNCNDSLGLALIYKCW	1	97.25%	Missing
256	VLNCNASLGLVLFYKCW	1	97.27%	Missing
257	ILNCNDSYGLALFYRCW	1	97.28%	Missing
258	ILNCNDSLGLALIYNCW	1	97.30%	Missing
259	ILNCNASDGVALFYKCW	1	97.32%	Missing
260	LLNCNDSLGLAPFYKCW	1	97.34%	Missing
261	ILNCNASLGIALIHKCW	1	97.36%	Missing
262	ILNCNDALGIALLYKCW	1	97.38%	Missing
263	ILNCDESIGIACFYKCW	1	97.40%	Missing
264	ILNCNDSLGIALIYRCF	1	97.42%	Missing
265	VLNCNESLGLALFYRCW	1	97.43%	Missing
266	ILNCNASLGLALFHKCW	1	97.45%	Missing
267	ILNFNASLGIALFYKCW	1	97.47%	Missing
268	ILNCNDSQGLALFYRCW	1	97.49%	Missing
269	ILNCNESLGIALLYSCW	1	97.51%	Missing
270	ILNCNESLGIALFYRCR	1	97.53%	Missing
271	ILDCNASLGVALFYKCW	1	97.55%	Missing
272	ILNCEDSLGIALIYKCW	1	97.57%	Missing
273	ILNCNDSIGIALMYKCW	1	97.58%	Missing
274	ILNCNDSLGLALLYHCW	1	97.60%	Missing
275	IPNCNESIGIALFYKCW	1	97.62%	Missing
276	ILNCNDTLGLALFYKRW	1	97.64%	Missing
277	IPNCNDSLGLALFYKCW	1	97.66%	Missing

278	VLNCNDSLGIALFYWCW	1	97.68%	Missing
279	ILNCNDSLGIALXYRCW	1	97.70%	Missing
280	ILNCNASLGVALFYKCF	1	97.72%	Missing
281	ILNCNDSPGLALFYKCW	1	97.73%	Missing
282	ILNCNDPIGIALFYKCW	1	97.75%	Missing
283	ILNCNASLGLAPFSKCW	1	97.77%	Missing
284	VLNCNDSLGIALVYQCW	1	97.79%	Missing
285	LLNCTP--------PCW	1	97.81%	Missing
286	LLNCNESLGLALFYKCW	1	97.83%	Missing
287	VLNCNASLGVALFYKCW	1	97.85%	Missing
288	ILNCNESLGLVLFYNCF	1	97.87%	Missing
289	ILNCNESLGFTLFYHCW	1	97.88%	Missing
290	ILNCNASHGIALFYKCW	1	97.90%	Missing
291	ILNCNASLGLALFNKCW	1	97.92%	Missing
292	ILNCNESFGIALFYKCW	1	97.94%	Missing
293	ILNCDDALGIALMYRCF	1	97.96%	Missing
294	ILNCNASLGLVLFYKCW	1	97.98%	Missing
295	ILNCNTSLGLALIYKCW	1	98.00%	Missing
296	VLNCNDTLGIALFYRCW	1	98.01%	Missing
297	ILNCNDSLGIALLYXCW	1	98.03%	Missing
298	VLNCNDSLGIALFYQCW	1	98.05%	Missing
299	ILNCNDSFGLALFYSCW	1	98.07%	Missing
300	ILNCNDTLGIALMYKCW	1	98.09%	Missing
301	VLNCNASLGLTLFYKCW	1	98.11%	Missing
302	ILNCNDSLGLALSYRCW	1	98.13%	Missing
303	ILNCNDSLGIALMYRCF	1	98.15%	Missing
304	ILNCNDTLGLALFARCW	1	98.16%	Missing
305	ILNCNDSIGIALLYRCW	1	98.18%	Missing
306	ILNCNASLGIALMYSCW	1	98.20%	Missing
307	ILNCNDSVGIALFYKCW	1	98.22%	Missing
308	ILNCHDSLGVALFYKCW	1	98.24%	Missing
309	ILSCNESLGLALIYKCW	1	98.26%	Missing
310	ILNCNGSLGIALFYRCW	1	98.28%	Missing
311	ILNCNDSIGIALFYMCW	1	98.30%	Missing
312	ILNCNDSLGVALLYNCW	1	98.31%	Missing
313	ILNCNASLGLTLFYKCW	1	98.33%	Missing
314	ILNCNDSLGFALFYACW	1	98.35%	Missing
315	ILNCDDSLGMALFYKCW	1	98.37%	Missing
316	ILNCNDSYGLALFYSCW	1	98.39%	Missing
317	ILNCNESIGMALFNACW	1	98.41%	Missing
318	ILNCNESLGLALFYKWW	1	98.43%	Missing
319	ILNCNDSLGLALIYRCW	1	98.45%	Missing
320	ILSCNDSLGLALLYKCW	1	98.46%	Missing

321	ILNCNESIGIALFNKCW	1	98.48%	Missing
322	ILNCNASLGIALFYSCW	1	98.50%	Missing
323	ILNCNDSLGIALFYECW	1	98.52%	Missing
324	LLNCNDSPGLALFYKCR	1	98.54%	Missing
325	ILDCKASLGLALLYKCR	1	98.56%	Missing
326	ILNCNDSLGIALFRKCW	1	98.58%	Missing
327	ILNCNDSIGMALFYRCW	1	98.60%	Missing
328	ILNCNASLGMALFYKCL	1	98.61%	Missing
329	ILNCNASIGIALFNKCW	1	98.63%	Missing
330	ILNCNASLGIALVYKCW	1	98.65%	Missing
331	VLNCNASLGIALFYKCW	1	98.67%	Missing
332	ILNCDESLGLALLYQCW	1	98.69%	Missing
333	ILNCFDSIGLALFARCS	1	98.71%	Missing
334	ILNCNDSIGIALFYNCW	1	98.73%	Missing
335	ILNCDESIGIALFYKCR	1	98.75%	Missing
336	ILNCNESLGIALIYRCW	1	98.76%	Missing
337	IRNCNESIRLALFYKCW	1	98.78%	Missing
338	ILNCNTSIGLALFYKCW	1	98.80%	Missing
339	ILNCNESHGLALFYKCW	1	98.82%	Missing
340	ILNCNDSLGIALFYNSW	1	98.84%	Missing
341	ILDCNESLGIALFYRCW	1	98.86%	Missing
342	ILNCNESLGTALFYKCW	1	98.88%	Missing
343	ILNCNASLGIALLYRCW	1	98.90%	Missing
344	VLNCNDSIGIALFYKCW	1	98.91%	Missing
345	ILNCNEYNRLALFYKCW	1	98.93%	Missing
346	ILNCNASLGLALIYQCW	1	98.95%	Missing
347	ILNCNDSYGIALFYNCW	1	98.97%	Missing
348	VLNCNDSLGIALIYKCW	1	98.99%	Missing
349	VLNCNESIELALFYKCW	1	99.01%	Missing
350	VLNCNDSLGVALFYRCW	1	99.03%	Missing
351	VLNCNDSIGIALIYKCW	1	99.04%	Missing
352	ILNCNDSLGIASFYRCW	1	99.06%	Missing
353	ILNCNESLGLALFYACW	1	99.08%	Missing
354	ILKCNDSLGIALFYKCW	1	99.10%	Missing
355	ILNCNDSIGIALIYSCW	1	99.12%	Missing
356	LLNCNDSLGLALFYRCW	1	99.14%	Missing
357	ILNCNDSLGLALFYRXW	1	99.16%	Missing
358	ILNCNESLGMALFYRCW	1	99.18%	Missing
359	ILNCNDTLGIXLLYKCW	1	99.19%	Missing
360	LLNCNGSLGLALFYKCW	1	99.21%	Missing
361	ILNCNESLDIALFYKCW	1	99.23%	Missing
362	IPNCNDSLGIALIYKCW	1	99.25%	Missing
363	VLNCNDSVGIALFYKCW	1	99.27%	Missing

364	ILNCNASHGVALLYKCW	1	99.29%	Missing
365	ILNCNTSHGIALIYKCW	1	99.31%	Missing
366	VLNCNDSLGLALFYSCW	1	99.33%	Missing
367	VLSCDDSLGIALFYKCW	1	99.34%	Missing
368	ILNCNDSLGLALLYXCW	1	99.36%	Missing
369	ILNCNASIGIALFYNCF	1	99.38%	Missing
370	ILNCNESLGJALFYKCW	1	99.40%	Missing
371	VLNCNDSLGLTLFYKCW	1	99.42%	Missing
372	ILNCNDSLGIALLYRCF	1	99.44%	Missing
373	ILHCNESIGIALFYKVW	1	99.46%	Missing
374	LLNCNDSLGLALFYKCR	1	99.48%	Missing
375	ILNCNDSIGIALFYQCW	1	99.49%	Missing
376	ILNCNDSLGIALFHRCW	1	99.51%	Missing
377	VLNCNDSLGLALFFRCW	1	99.53%	Missing
378	VLNCNDSLGISLFNRCF	1	99.55%	Missing
379	ILNCDDSIGLALFYKCW	1	99.57%	Missing
380	ILNCNTSLGIALFSNCW	1	99.59%	Missing
381	VLNCNDSLGFVLFYNCW	1	99.61%	Missing
382	ILNCNDTLGVALFYKCW	1	99.63%	Missing
383	ILNCNDSLGLAVFYKCW	1	99.64%	Missing
384	ILNCNDSLGIVLFYNCW	1	99.66%	Missing
385	ILNCNDSLGLALFYK--	1	99.68%	Missing
386	ILNCNDSIGIALIYKCG	1	99.70%	Missing
387	ILNCNDSLGIALF-ICW	1	99.72%	Missing
388	ILNCNDSLGLALFSKCW	1	99.74%	Missing
389	ILNCNDSLGMVLFYKCW	1	99.76%	Missing
390	ILNCHDSLGIALMYKCW	1	99.78%	Missing
391	ILNCSDTLGIALFYKCW	1	99.79%	Missing
392	ILNCNDSLGIALMYRCW	1	99.81%	Missing
393	ILNCNDSLGISLFYMCW	1	99.83%	Missing
394	ILNCNDSLGIALFYGCW	1	99.85%	Missing
395	ILNCNDSFGLALFYRCW	1	99.87%	Missing
396	ILNCNASLGMALFYKCW	1	99.89%	Missing
397	VLNCNDSLGLGLFYRCW	1	99.91%	Missing
398	ILNCNDSLGITLFYTCW	1	99.93%	Missing
399	ILNCNDSLGIALFYKCF	1	99.94%	Missing
400	ILNCNTSLGLALFYQCW	1	99.96%	Missing
401	ILNCNESIGVALLYKCW	1	99.98%	Missing
402	ILNCNDSLGISLIYRCW	1	100.00%	Missing
